# Feasibility of Using a Prosthetic-Based Impression Template to Improve the Trueness and Precision of a Complete Arch Digital Impression on Four and Six Implants: An In Vitro Study

**DOI:** 10.3390/ma13163543

**Published:** 2020-08-11

**Authors:** Marco Tallarico, Aurea Immacolata Lumbau, Roberto Scrascia, Gianluca Demelas, Franco Sanseverino, Rocco Amarena, Silvio Mario Meloni

**Affiliations:** 1 School of Dentistry, University of Sassari, 07021 Arzachena, Italy; alumbau@uniss.it (A.I.L.); gianlucademelas7@gmail.com (G.D.); melonisilviomario@yahoo.it (S.M.M.); 2 Private Practice, 74100 Taranto, Italy; roberto.scrascia@gmail.com; 3 Private Practice, 40100 Bologna, Italy; ls@newancorvis.eu; 4 Private Practice, 40100 Bologna, Italy; rd2@newancorvis.eu

**Keywords:** intraoral scanners, dental implants, fully edentulous, precision, trueness

## Abstract

Background: Intraoral scanners (IOSs) in implantology represent a viable approach for single teeth or partial arches. However, when used for complete edentulous arches or long-span edentulous areas, it has been demonstrated that there is a need for improvement of IOS-related techniques. Therefore, the aim of this in vitro study was to assess the trueness and precision of a complete arch digital impression on four and six implants taken with or without a customized, prosthetic-based impression template. Materials and Methods: Two experimental models were prepared, representative of a complete edentulous mandible restored with four and six implants with built-in scan abutments. Models were scanned with (test group, TG) or without (control group, CG) the prosthetic-based impression template. Eight scans were taken for each model. The time needed to take impressions, error, trueness, and precision were evaluated. A statistical analysis was performed. Results: In the case of four implants, the time needed for the impression was 128.7 ± 55.3 s in the TG and 81.0 ± 23.5 s in the CG (*p* = 0.0416). With six scan abutments, the time was 197.5 ± 26.8 and 110.6 ± 25.2 s in the TG and CG, respectively (*p* = 0.0000). In the TG, no errors were experienced, while in the CG, 13 impressions were retaken due to incorrect stitching processes. In the four-implant impression, the mean angle deviation was 0.252 ± 0.068° (95% CI 0.021–0.115°) in the CG and 0.134 ± 0.053° (95% CI 0.016–0.090°) in the TG. The difference was statistically significant (*p* = 0.002). In the six-implant impression, the mean angle deviation was 0.373 ± 0.117° (95% CI 0.036–0.198°) in the CG and 0.100 ± 0.029° (95% CI 0.009–0.049°) in the TG (*p* = 0.000). In the TG, there were no statistically significant differences in the mean angle deviation within the group (*p* > 0.05), but there were in the CG. A colorimetric analysis showed higher deviations from the original model for the six-implant impression without a prosthetic template. Conclusions: Although all of the impressions exhibited deviation from the original model in the range of clinical acceptability, the prosthetic-based impression template significantly improved the trueness and precision of complete edentulous arches rehabilitated with four or six implants, making the complete arch digital impression more predictable.

## 1. Introduction

In the last few years, intraoral scanners (IOSs) have represented a viable approach for the diagnosis, planning, and execution of treatments [1,2,3,4]. One of the major contributions to the rapid spread of digital impressions is the fact that IOSs have been demonstrated to perform within the same range of accuracy as conventional impressions when used for short-span areas (single teeth or partial arches) [2,5]. This allows digital models to reach the high accuracy needed to guarantee the proper fit of dental restorations [6]. Moreover, when compared with analog impressions, digital technologies offer several benefits, such as patient acceptance, time efficiency, direct visualization of the impression, and quick and easy repeatability [2,3,4,5]. Last but not least, IOSs can be used in combination with other digital technologies, such as computer-aided design/computer-aided manufacturing (CAD/CAM) technologies, for chairside production, or in combination with cone beam computed tomography (CBCT), to make computer-guided surgery easier [7,8,9,10]. Nevertheless, when used for complete edentulous arches or long-span edentulous areas, it has been demonstrated that there is a need for improvements of IOS-related techniques, in order to reach the same levels of accuracy achieved with conventional impressions [11,12,13,14,15,16,17,18]. Whilst powdered intraoral scanners showed promising results, they have been withdrawn from the market [19]. Moreover, at the time of writing this article, there were no randomized controlled trials (RCTs) proposing novel techniques or materials to improve the accuracy of implant-based digital impressions for complete edentulous arches.

For the latter, the accuracy is defined with trueness and precision. Trueness can be evaluated by comparing the master model (original geometry) with the digitized impression. Moreover, precision can be obtained by an intragroup comparison of the digitized models [17,18].

In 2017 [19] and 2018 [20], Tallarico et al. published a fully digital workflow to rehabilitate edentulous patients. In order to improve the accuracy of the digital impressions, a novel, prosthetic-based impression template, made by virtual planning, was presented. This prosthetic template was customized by maintaining the original tooth design, but including four windows, to allow the screwing of scan abutments, so that the impression could be matched with the initial planning.

The aim of the present in vitro comparative study was to assess the trueness and precision of complete arch digital impressions on four and six implants taken with or without the prosthetic-based impression template. The null hypothesis of this research was that there are no statistically significant differences between different impression techniques.

## 2. Materials and Methods

**Figure 1:**
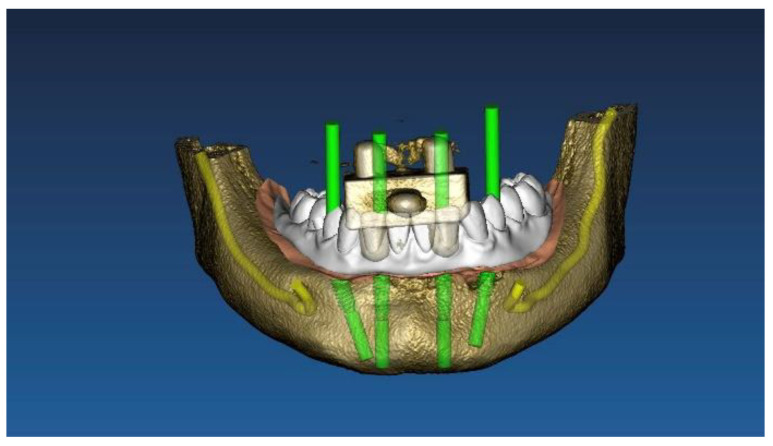
Virtual implant planning of four implants according to the All-on-4 protocol.

**Figure 2:**
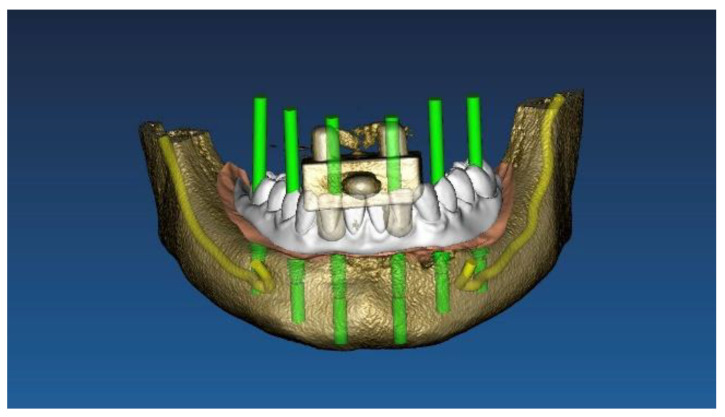
Virtual implant planning of six straight implants.

**Figure 3:**
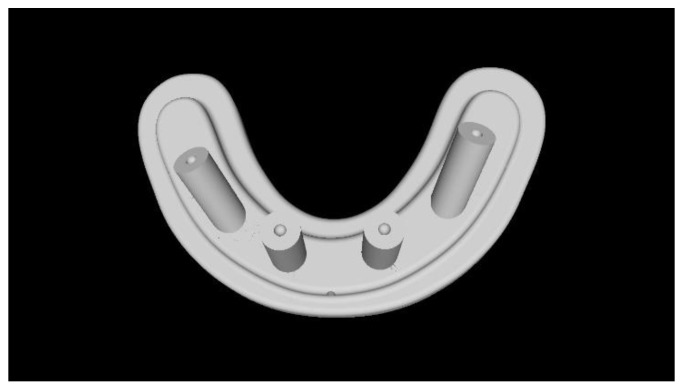
Computer-aided design of the model with four implants.

**Figure 4:**
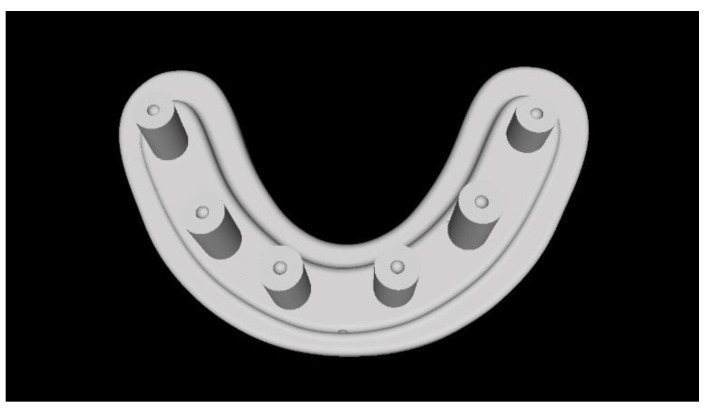
Computer-aided design of the model with six implants.

**Figure 5:**
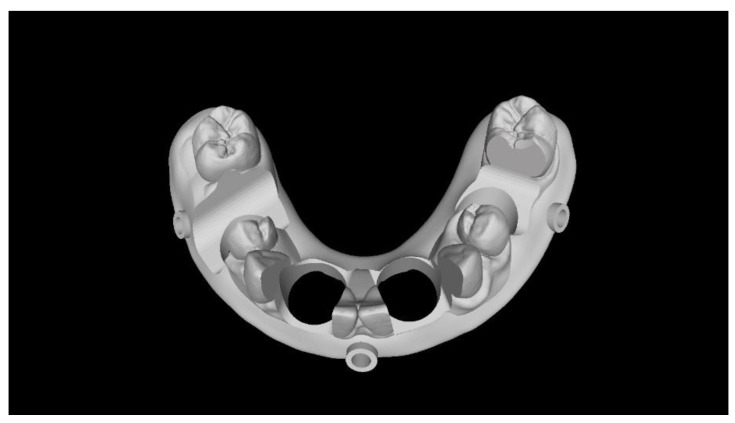
Computer-aided design of the prosthetic template for the four-implant impression.

**Figure 6:**
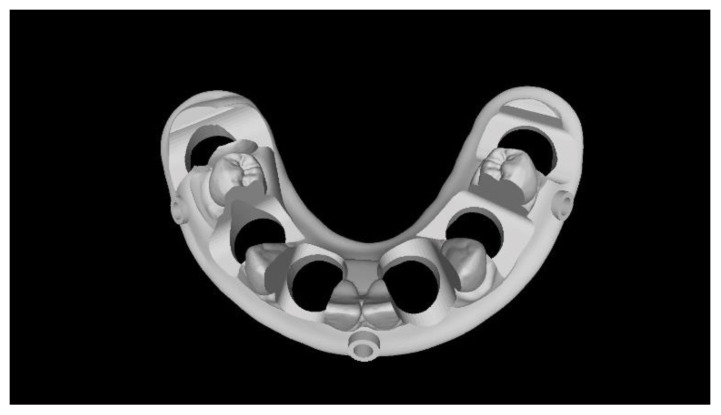
Computer-aided design of the prosthetic template for the six-implant impression.

**Figure 7:**
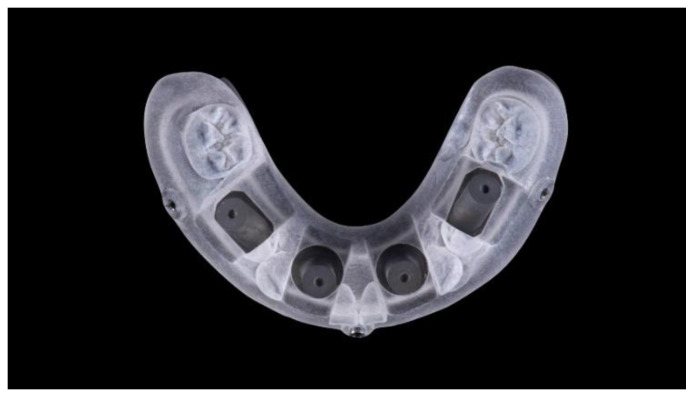
Titanium model (four implants) with a prosthetic template screwed.

**Figure 8:**
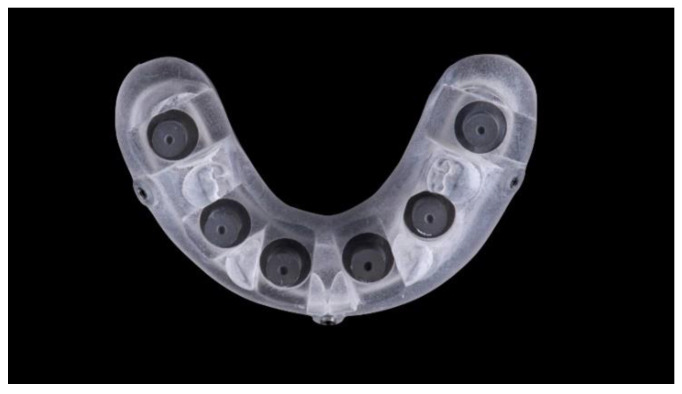
Titanium model (six implants) with a prosthetic template screwed.

Two different virtual implant plans of the same, real complete edentulous mandibular arch were performed with four and six implants, respectively (RealGUIDE5, version 5.0, 3DIEMME srl, Cantù, Italy). In the four-implant plan, the implants were placed according to the All-on-4 protocol [21], tilting the distal implants by 30° (Figure 1). In the six-implant plan, all of the implants were placed according to a pre-established prosthetic setup, straight and parallel between them (Figure 2). Then, virtual implant positions were exported and two experimental models were designed (Rhino 6, Rhinoceros, McNeel Europe, Barcelona, Spain) and milled in titanium grade 5 (New Ancorvis SRL, Calderara di Reno (BO), Italy). The decision to mill the models in titanium was made in order to create stable and durable models with an opaque, micro abraded surface (no scanning spray required), avoiding a risk of bias. Both models were derived from the same prosthetic setup, simulating a complete denture. The first model was designed by placing the four implants according to the All-on-4 protocol and with built-in scan abutments (Figure 3), while the second model was created by placing six straight implants, with the same in-build scan abutments (Figure 4). Each scan abutment was designed to be 10 mm in length and 4 mm in diameter. Two prosthetic-based impression templates (prosthetic template) to be used during the complete-arch digitalization were designed (RealGUIDE5) and then prepared for printing (Materialise Magics 24, Materialise, Leuven Belgium). At this point, four (Figure 5) or six (Figure 6) windows were created in the prosthetic templates (Materialise Magics 24) to accommodate the scan abutments, ensuring accurate fitting of the template (Figure 7 and Figure 8). The windows were created by subtracting solid shapes from the original STL files, without compromising the stability of the prosthetic template and maintaining at least five teeth that acted as landmarks between scan abutments. To fix the template to the titanium model, three pre-planned screws were used. Finally, the templates were printed using the ProJet MJP 2500 Plus with VisiJet M2R-CL (3D System Inc., Rock Hill, SC, USA).

Models with a respective prosthetic template were immobilized using a customized metal base and then manually digitalized using the Medit i500 intraoral scanner (Medit Corp., Seoul, Korea) with level-2 filtering and a 17.0 mm depth, following the manufacturer’s guidelines. The right side of each model was digitalized first (Medit Link software version 2.2.2.753, Medit Corp.). An expert operator (MT) started placing the camera on the most right distal scan abutment and then started digitalizing the occlusal surface by pushing the button on the scanner. Following this, the process proceeded from the right to the left side of the arch by rolling the camera to the buccal and lingual areas of the models, until the complete occlusal surface had been digitized. According to the protocol, when reaching the anterior area, zigzag movements between the lingual and buccal areas, centered around the central edge, were performed to extend the anterior digitalized zone, making the remaining areas easier to match. Once the opposite side had been scanned, the lingual and finally buccal sides were digitalized. To move from the occlusal to lingual zone, the tip of the scanner was tilted at around 45° toward the lingual side and then moved to the opposite side. Once the lingual side was completed, the tip of the scanner was tilted to the buccal site and moved from there to the opposite area. Before processing the impression, the scan data was checked. In the case of incomplete surfaces, the tip of the scanner was positioned in the area to complete the data. Each model (test group, four and six scan abutments) was digitalized eight times. After that, the prosthetic templates were removed by unscrewing the fixing screws, and the models were digitalized individually eight times each, following the same aforementioned protocol. The scanner was calibrated before any impression, according to the manufacturer’s instructions. All of the impressions were processed by the software and then exported in Standard Triangle Language (STL) format, in a share folder, using a file hosting service (Dropbox, Inc., San Francisco, CA, USA).

The outcome measures were as follows:

The time (seconds) needed to take impressions was automatically calculated using the digital chronometer of the software.

Any error dictating impression retaking, such as distortion, an incorrect stitching process, or failure due to overlap was noted.

**Figure 9:**
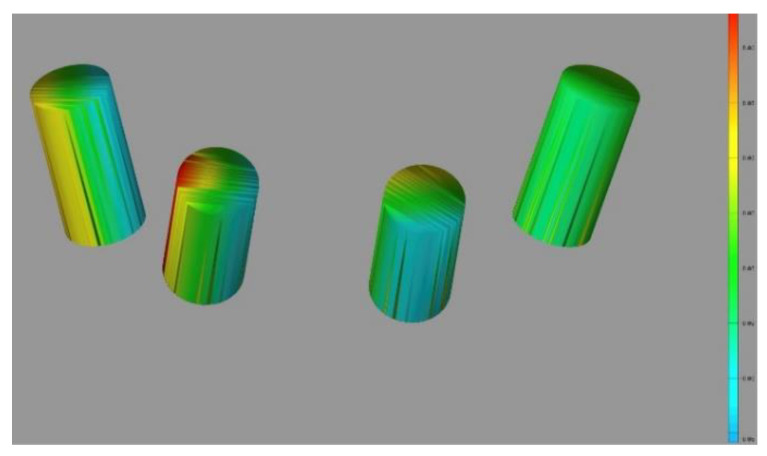
Colorimetric analysis (GOM inspect) of the four-implant impression using the prosthetic template.

**Figure 10:**
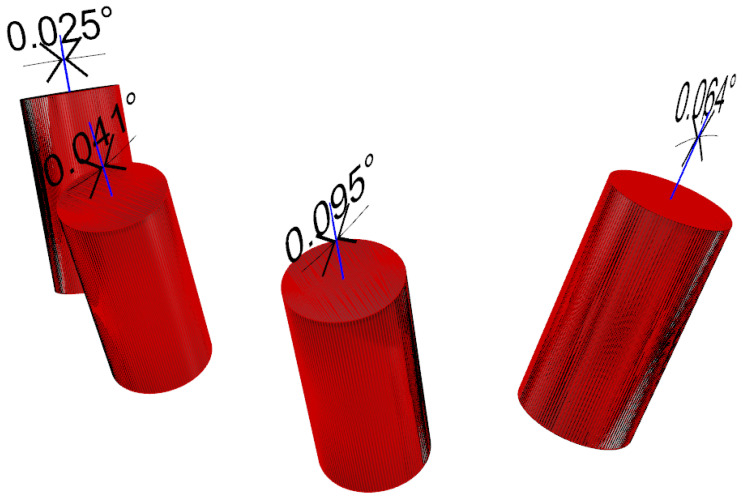
Angular deviation between the digitalized and original scan abutment position (four-implant impression), calculated along the long axis of each scan abutment after superimposition.

**Figure 11:**
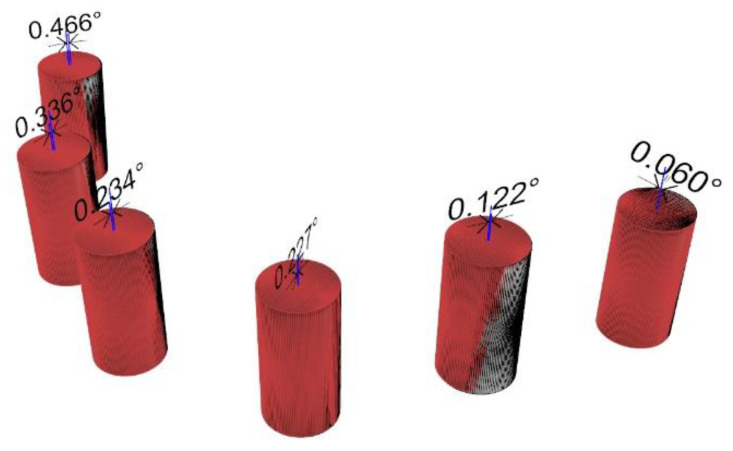
Angular deviation between the digitalized and original scan abutment position (six-implant impression), calculated along the long axis of each scan abutment after superimposition.

Trueness and precision were established by measuring the difference in angle between the original (the truth) and digitalized scan abutment position. The postoperative STL file, derived from the intraoral scan, was geometrically aligned with the original STL file, by automated image registration, using the maximization of mutual information (Optical RevEng4.0, Open Technologies, Rezzato (BS), Italy). The trueness was assessed using GOM Inspect Professional (GOM, Braunschweig, Germany) after aligning the experimental models and the digitalized impressions by using best-fit algorithms. After superimposition, the deviations between selected surfaces were assessed and the qualitative analyses were presented using colorimetric scale measurements. Deviations at tolerance levels from 0.01 to 0.05 mm were analyzed (Figure 9). The precision was assessed as the angular deviation between the digitalized and original scan abutment position, calculated along the long axis of each scan abutment (Rhino 6) after library alignment (Exocad Plovdiv, Exocad GmbH, company, Darmstadt, Germany). An expert biomedical engineer performed all of the measurements (RA) (Figure 10 and Figure 11).

Statistical analyses were performed using NUMBERS, version 10.0 (6748) (Apple Inc., Cupertino, CA, USA) and online calculators [22,23,24,25]. Mean values, standard deviations (SD), and the 95% confidence interval (CI) were calculated. Comparisons between groups for continuous outcomes (time and accuracy) were made by paired tests, in order to detect any changes in the impression accuracy. One-way analysis of variance (ANOVA) was conducted to determine the effect of the scan abutment position and angulation on the overall accuracy. Differences in the proportion of errors during the impression (dichotomous outcomes) were compared between groups using the 2 × 2 Fisher exact test. Impression was the statistical unit. The statistical significance was set at 0.05. Based on the authors’ knowledge, there are no similar studies in the scientific literature. Therefore, an a priori sample size calculation was not performed. We decided to scan each model eight times, according to or better than previous in vitro studies comparing IOSs [3,4]. A post hoc analysis of continuous variabilities (mean angular deviation between groups) was performed by calculating the effect size (Cohen’s d) and giving the means of each group, number of samples (*n* = 16), and alpha value (0.05).

## 3. Results

**Figure 12:**
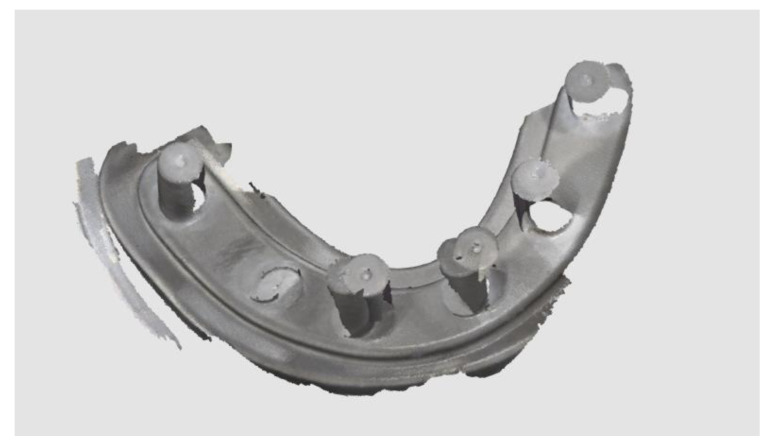
Incorrect stitching process experienced during digital impression of the six-implant impression without the prosthetic template.

The time needed to take the impressions with four scan abutments was 128.7 ± 55.3 and 81.0 ± 23.5 s in the test and control group, respectively. The differences were statistically significant (*p* = 0.0416). The time needed to take the impressions with six scan abutments was 197.5 ± 26.8 and 110.6 ± 25.2 s in the test and control group, respectively. The differences were statistically significant (*p* = 0.0000). In the test group, no errors were experienced during impression taking, while in the control groups, 13 impressions were retaken (11 impressions with six scan abutments and two impressions with four scan abutments) due to incorrect stitching processes (Figure 12). The differences were statistically significant when six scan abutments were digitalized (*p* = 0.008), but not when four scan abutments were digitalized (*p* = 0.447).

When four scan abutments were digitalized, the mean angle deviation was 0.252 ± 0.068° (95% CI 0.021–0.115°) in the control group and 0.134 ± 0.053° (95% CI 0.016–0.090°) in the test group. The difference was statistically significant (0.118 ± 0.077°; 95% CI 0.024–0.131°; *p* = 0.002). When six scan abutments were digitalized, the mean angle deviation was 0.373 ± 0.117° (95% CI 0.036–0.198°) in the control group and 0.100 ± 0.029° (95% CI 0.009–0.049°) in the test group. The difference was statistically significant (0.273 ± 0.111°; 95% CI 0.034–0.188°; *p* = 0.000).

In the test group, there were no statistically significant differences in the mean angle deviation within groups (four scan abutments, *p* = 0.391 and six scan abutments, *p* = 0.372). In the control group, there were statistically significant differences in the mean angle deviation within groups. In the case of four digitalized abutments, a higher angle deviation was found in the last left scan abutment (position 34, 0.510 ± 0.191°, *p* = 0.00005). In the case of six digitalized abutments, a higher angle deviation was found in the first scan abutment (position 46, 0.616 ± 0.306°; *p* = 0.00766).

Post hoc power analysis demonstrated power in a range of 94.9 to 100% in the case of four and six implants, respectively. The mean angular deviation values between groups are summarized in Table 1.

Colored representation showing the smallest deviation using the surgical template in the four-implant model. Nevertheless, similar deviations when observed without surgical templates for the same four-implant model. Deviations of six-implant models were larger. However, the worst results were observed without the surgical templates.

## 4. Discussion

This in vitro study was designed to provide preliminary data on whether it would be more advisable to use the proposed prosthetic template when a digital impression was taken to fabricate an implant-supported complete arch restoration. The results of the present study demonstrated that the prosthetic-based impression template significantly improved the trueness and precision of complete edentulous arches rehabilitated with four or six implants. Hence, the null hypothesis of this research was rejected in favor of the alternative hypothesis of differences.

A precise impression still remains as one of the most important steps for fabricating dental-tooth and implant-supported restorations with an adequate fit, avoiding risks of mechanical and biological complications [13,14,26]. Digital impressions have been suggested as a valid alternative to conventional impressions for partial arch rehabilitation [2], while complete-arch impressions still remain a challenge when IOS devices are used [13,14,15,16]. Intraoral scanning systems are not without technology-related errors. Most of the drawbacks in complete-arch impressions could be due to a lack of fixed references. It has been suggested that the longer the scanning field, the more stitching processes with possible errors are presented [5].

To reduce this possible drawback, in the present study, the proposed prosthetic template, derived from the original tooth-setup by duplicating the complete denture by using an IOS or desktop scanner, was used. The main benefit of the prosthetic template is its ability to provide fixed references between scan abutments, in order to improve the readability of the IOS, even in complex scenarios, making digital impressions for complete-arches more predictable. A second benefit is that it allows matching between the implant positions and the original prosthetic setup. This allows transferring the match between the prosthetic volume (esthetic and function) of the setup and the final implant position to be transferred. The vertical dimension of occlusion and centric relation are also transferred.

To the best of the authors’ knowledge, no previous study has compared the accuracy of a complete arch digital impression on four and six implants with and without a prosthetic template.

Data from the present study demonstrate that the overall accuracy of digital impressions is statistically significantly higher when a prosthetic template is used. The mean angular deviation experienced when using the prosthetic template was 0.100 and 0.134° with six and four implants, respectively. These values correspond to a linear deviation of about 88 and 119 µm. Previous clinical studies showed that the threshold for a clinically acceptable fit implant-supported fixed prosthesis is within a range of 59–200 μm [27,28,29,30,31]. On the other hand, when the surgical templates were not used, the present research found a mean angular deviation of 0.252 and 0.373° with four and six implants, respectively. These values correspond to a linear deviation of about 224 and 331 µm that appear to be in disagreement with what has been previously reported. The aforementioned findings suggest that the complete arch digital impression still remains a challenge and efforts must be performed to increase the accuracy. To date, it is the authors’ opinion that a try-in with an aluminum framework is mandatory before the manufacturing of the definitive prosthesis. Nevertheless, data from the present study demonstrated that when using the prosthetic template, the mean accuracy was statistically significantly higher. This makes the digital impressions taken with the prosthetic template more predictable. In fact, even if the overall time needed to take the impressions was lower in the control group, 13 impressions were retaken due to incorrect stitching processes, making the impressions without the prosthetic template more time consuming. In the present study, the time needed to properly set the prosthetic template was not calculated because it was fixed to the model for all of the impressions. In a real practice, the prosthetic template can be fixed using the same anchor pins planned for the surgical implant placement or can be fixed in occlusion with a flowable resin composite, extending the time needed to take the impression.

The present study failed to find any statistically significant differences in the mean angle deviation within groups when the prosthetic template was used. The same results were not experienced when the prosthetic template was not used, with a higher angle deviation in the last (distal) abutments, in both four- and six-implant impressions. This means that when using the prosthetic template, the accuracy of each scan abutment is predictable regarding the implant position and angulation. This could be helpful for reducing the learning curve, making the impression not operator-dependent.

In 2017 [19] and 2018 [20], Tallarico et al. published a customized prosthetic template with the purpose of improving the accuracy of the digital impressions for implant-supported complete-arch restorations. After that, some case reports describing similar concepts were proposed. In 2019, Venezia et al. [32] presented the evolution of the previously published BARI technique, which allowed the digital transfer of the maxillary–mandibular relationship, from the complete denture to the implant-supported 3D-printed hybrid prosthesis. For the definitive impressions, prosthetic stents derived from the original plan were used. At the beginning of 2020, Ahmed et al. [33] published a digital scanning and maxillomandibular relationship workflow for maxillary complete-arch implant-supported restoration. Even in this case, a custom scanning device was used during the complete-arch intraoral scan. However, the present research is the only study evaluating the trueness and precision of the prosthetic template.

The prosthetic template can be fabricated starting from the original prosthetic setup used to virtually plan the implants or duplicating the existing complete denture of the patient [34,35,36,37]. The prosthetic template includes a certain number of windows to allow screwing of the scan abutments, as well as teeth surfaces that act as reference points to improve the accuracy of IOS and later match the digitalized impression with the initial plan [37]. There are some potential limitations for the use of the prosthetic template, such us the fixation method of the template, as well as its costs. When used in combination with a guided surgery, the prosthetic template can be designed with the same anchor pins used to stabilize the surgical guide [29,30]. This allows the prosthetic template to be stabilized during impression taking. On the other hand, when used after surgery, the prosthetic template can be stabilized in occlusion, fixing the scan abutments to the prosthetic template, avoiding possible negative effects due to template movements during scanning, and leading to the recording of an accurate impression that can be taken chairside or extra-orally. All of these methods have been published in previous clinical case reports [19,20], showing promising results. Regarding the overall costs, looking at the whole scenario, the prosthetic template allows the overall treatment time to be reduced, avoiding vertical dimension and occlusion recording. Considering this, the overall cost could even be reduced.

The main limitation of this research was the in vitro nature of the study design. The in vitro evaluation may not have fully simulated the clinical practice environment or condition [38,39], such us the natural gingiva. However, it is highly likely that the benefits of the prosthetic template can even exceed the positive results of the present study. The accuracy of the IOS required as many reference points as possible. Moreover, it can be affected by the presence of saliva, such as movement of the soft-tissue area. The challenge of taking digital impressions of edentulous mandibular arches remains in the elasticity of gingiva. To partially overcome this drawback, artificial gingiva could be used. Nevertheless, in the present study, artificial gingiva was not used in order to avoid possible bias due to its possible shift or detachment. The prosthetic template can also be used extra-orally after fixing the scan abutment on its surfaces. Of course, the results of the present study require confirmation through further clinical studies, even if the clinical report suggests encouraging results.

## 5. Conclusions

Although all of the impressions showed a mean deviation from the original model in the range of clinical acceptability, the prosthetic-based impression template significantly improved the trueness and precision of complete edentulous arches rehabilitated with four or six implants. Due to the limitations of in vitro studies, further in vitro studies are needed to confirm these preliminary results.

## Figures and Tables

**Table 1:** Mean angular deviation between groups. Data are presented in degrees (°) as the mean ± SD (95% CI).

	Test	Control	Difference	*p*-Value
Four	0.134 ± 0.053 (0.016–0.090)	0.252 ± 0.068 (0.021–0.115)	0.118 ± 0.077 (0.024–0.131)	0.002
Six	0.100 ± 0.029 (0.009–0.049)	0.373 ± 0.117 (0.036–0.198)	(0.273 ± 0.111 (0.034–0.188)	0.000

One-way analysis of variance (ANOVA) tables that contain all relevant information from the observation data set, including sums of squares, mean squares, degrees of freedom, and *F*- and *p*-values.
